# MicroRNAs in spinal cord injury: A narrative review

**DOI:** 10.3389/fnmol.2023.1099256

**Published:** 2023-02-02

**Authors:** Chunjia Zhang, Zuliyaer Talifu, Xin Xu, Wubo Liu, Han Ke, Yunzhu Pan, Yan Li, Fan Bai, Yingli Jing, Zihan Li, Zehui Li, Degang Yang, Feng Gao, Liangjie Du, Jianjun Li, Yan Yu

**Affiliations:** ^1^School of Rehabilitation, Capital Medical University, Beijing, China,; ^2^Department of Spinal and Neural Functional Reconstruction, China Rehabilitation Research Center, Beijing, China; ^3^China Rehabilitation Science Institute, Beijing, China; ^4^Center of Neural Injury and Repair, Beijing Institute for Brain Disorders, Beijing, China; ^5^Beijing Key Laboratory of Neural Injury and Rehabilitation, Beijing, China; ^6^School of Rehabilitation Sciences and Engineering, University of Health and Rehabilitation Sciences, Qingdao, Shandong Province, China; ^7^Cheeloo College of Medicine, Shandong University, Jinan, Shandong Province, China; ^8^Department of Orthopedics, Qilu Hospital of Shandong University, Jinan, Shandong Province, China

**Keywords:** microRNA, biomarkers, spinal cord injury, neuroinflammatory response, pathophysiological mechanisms, therapeutic targets

## Abstract

Spinal cord injury (SCI) is a global medical problem with high disability and mortality rates. At present, the diagnosis and treatment of SCI are still lacking. Spinal cord injury has a complex etiology, lack of diagnostic methods, poor treatment effect and other problems, which lead to the difficulty of spinal cord regeneration and repair, and poor functional recovery. Recent studies have shown that gene expression plays an important role in the regulation of SCI repair. MicroRNAs (miRNAs) are non-coding RNA molecules that target mRNA expression in order to silence, translate, or interfere with protein synthesis. Secondary damage, such as oxidative stress, apoptosis, autophagy, and inflammation, occurs after SCI, and differentially expressed miRNAs contribute to these events. This article reviews the pathophysiological mechanism of miRNAs in secondary injury after SCI, focusing on the mechanism of miRNAs in secondary neuroinflammation after SCI, so as to provide new ideas and basis for the clinical diagnosis and treatment of miRNAs in SCI. The mechanisms of miRNAs in neurological diseases may also make them potential biomarkers and therapeutic targets for spinal cord injuries.

## 1. Introduction

Spinal cord injury (SCI) is a serious nervous system disease. In addition to limitations in motor and sensory function, patients with SCI also have dysfunction of multiple systems. This impairment leads to a severe deterioration in the quality of life of patients, increasing the disability rate of SCI and the mortality rate of SCI (Giger et al., 2010; Yan et al., 2012). About 90% of SCI is caused by trauma (Alizadeh et al., 2019).

**Figure 1:**
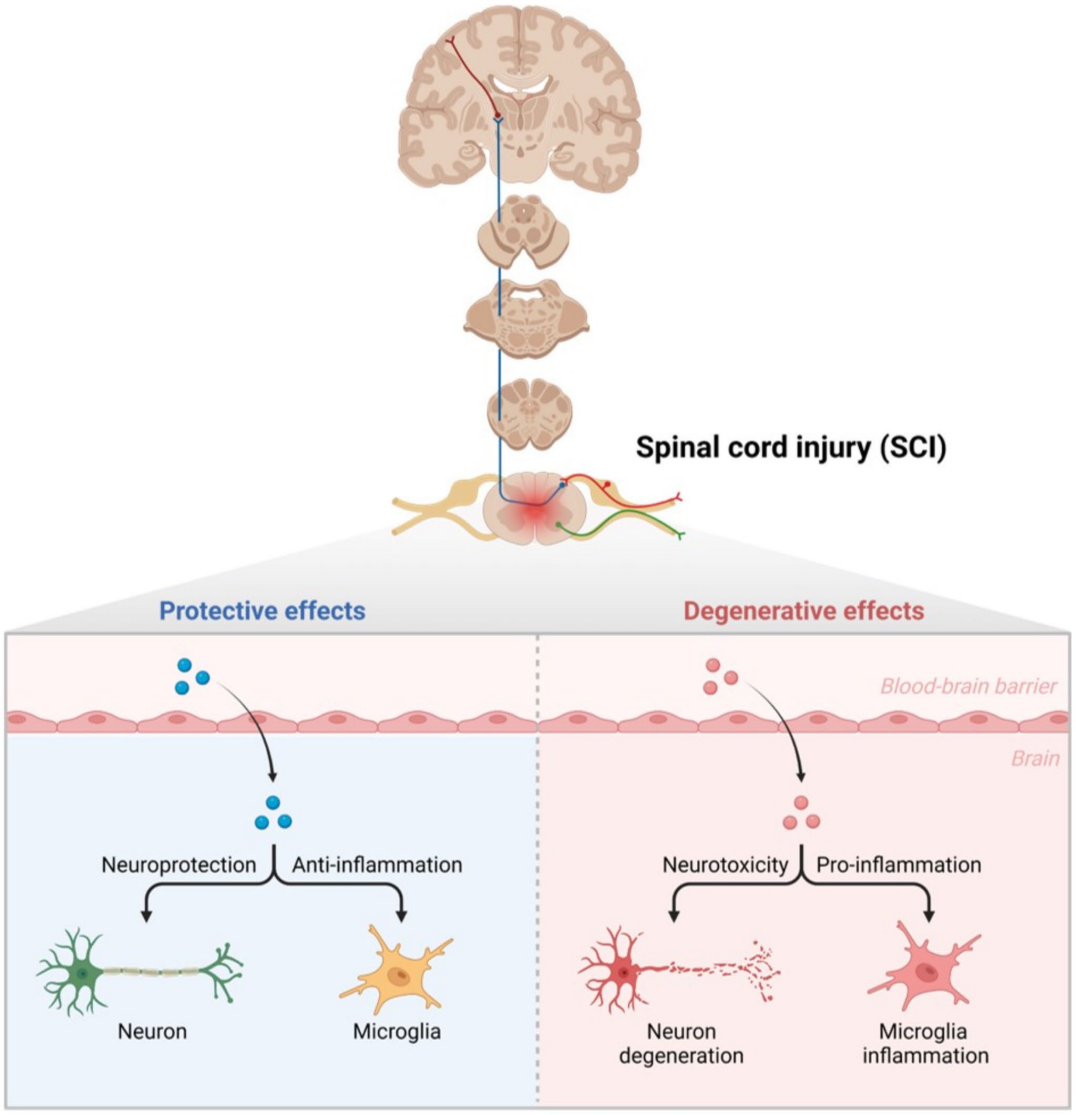
Glial cells in secondary injury after SCI. Normally, neurons play a protective role, and microglia resist the inflammatory response. Once SCI occurs, the blood–brain barrier is destroyed, leading to neuronal degeneration under the effect of neurotoxicity, and microglia cells play a pro-inflammatory role.

Spinal cord injury includes primary and secondary stages after injury. The primary stage includes the destruction of nerves and axons, hemorrhaging, and the destruction of glial membranes (Couillard-Despres et al., 2017; Anjum et al., 2020). Spinal cord compression intensity and severity are highly dependent on the initial injury. In addition to neurological, physiological, and biochemical changes, microglia and astrocytes are also affected (Figure 1). As cytotoxicity, reactive oxygen concentrations, and glutamate levels continue to rise, secondary damage is further exacerbated. The main clinical manifestations of secondary injury are vascular injury, ischemic edema, electrolyte disorders, inflammatory reactions, oxidative stress, cytotoxicity, apoptosis, glial scar formation, and Wallerian degeneration (Dimitrijevic et al., 2015; Anjum et al., 2020). After the primary stage, the body enters the secondary injury stage, including acute, subacute, and chronic phases. Ion imbalance, edema, necrosis, vascular injury, and inflammation are the main factors of the acute phase. The subacute stage mainly includes Wallerian degeneration, neuronal apoptosis, and axonal demyelination. After entering the chronic phase, axon remodeling and glial scar formation lead to fiber bundle disorders (Dumont et al., 2001; Bareyre and Schwab, 2003; Di Giovanni et al., 2003). Most studies and clinical interventions focus on the chronic stage. Spinal cord injury has the characteristics of great harm, high incidence, limited clinical diagnosis, and difficult treatment. X-ray, CT, MRI, lumbar puncture cerebrospinal fluid examination, Neurophysiological examination, etc., all belong to the physical examination of clinical diagnosis of SCI. Spinal MRI is the gold standard for evaluating spinal instability, spinal canal invasion, and intramedullary nerve structure injury caused by mechanical trauma. It plays an important role in the clinical diagnosis of SCI (Ellingson et al., 2014; Kumar and Hayashi, 2016). In clinical practice, MRI is often used to show the abnormal conditions inside and outside the medullary and make a preliminary judgment on the degree of injury (spinal cord compression, extent of disk herniation, ligament instability near the injury site; Kumar and Hayashi, 2016; Shah and Ross, 2016). However, conventional spinal MRI cannot provide or predict the association between adjacent spinal segments and degenerative changes in the brain, exploring the plasticity of the brain and spinal cord (Freund et al., 2019). This limitation has driven the development of subsequent quantitative MRI tests (magnetization transfer, magnetic resonance relaxation imaging, diffusion imaging, etc.). It also promotes the exploration and research of conventional MRI markers, neuroimaging biomarkers and effective biomarkers (Koskinen et al., 2013; Jirjis et al., 2016; Seif et al., 2018; Ziegler et al., 2018). MicroRNAs (MiRNA), as key regulatory factors in transcriptional regulation of gene expression changes in nervous system diseases, have been gradually paid attention to and studied by scholars. The direct contact between cerebrospinal fluid and blood in the central nervous system is considered an important source of biomarkers (Badhiwala et al., 2018; Baichurina et al., 2021). The current clinical treatment of SCI mainly includes emergency surgery in the acute phase, drug therapy, and rehabilitation therapy in the chronic phase (Furlan et al., 2016; Liu et al., 2018). However, suitable solutions for the problems of nerve regeneration and functional repair in patients with SCI have not been found, and there are no effective treatment plans. Some studies have tried to explore other interventions to treat SCI more accurately and effectively with the help of exosomes, cell transplantation, and biological scaffolds (Li et al., 2017; Lien et al., 2019; Mohammadshirazi et al., 2019).

## 2. MicroRNAs

**Figure 2:**
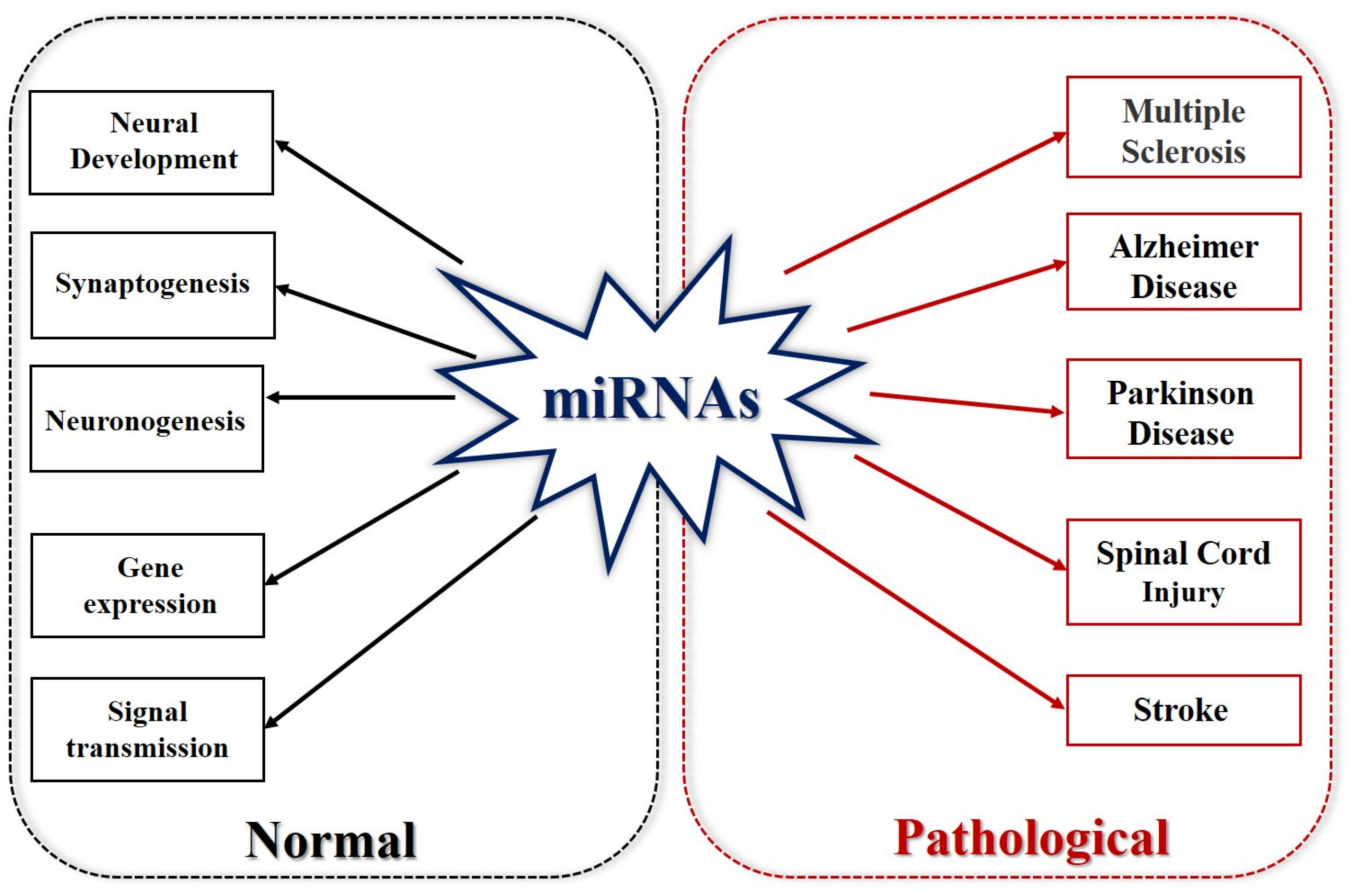
MicroRNAs in normal and pathological states. Under normal circumstances, miRNA is involved in neural development, synaptogenesis, neurogenesis, gene expression, and signal transmission. In its pathological state, miRNA is involved in multiple sclerosis, Alzheimer’s disease, Parkinson’s disease, SCI and stroke.

Non-coding RNA refers to RNA that does not encode proteins. These include rRNA, tRNA, snRNA, snoRNA, microRNA, and other RNAs with known functions, as well as RNAs with unknown functions (Shi et al., 2022). MicroRNAs (miRNAs) are a group of endogenous non-coding single-stranded RNAs that regulate gene expression (Ho et al., 2022). Short RNA molecules, microRNAs contain 19–25 nucleotides. The 3′-untranslated region (3′-UTR) of a target RNA allows a miRNA to target hundreds of other mRNAs. It can cause mRNA degradation or inhibit mRNA expression at the transcriptional level. miRNAs can regulate protein synthesis, gene expression, and thus affect various diseases and growth and development processes. miRNAs play equally important roles in maintaining morphological stability, axonal morphology and plasticity of cells in the nervous system (Bartel, 2004; Ning et al., 2014). The total number of human miRNAs is about 800, and they act on 30% of protein-coding genes (Bentwich et al., 2005; Li et al., 2009). miRNAs are associated with many allergic diseases, such as eczema, allergic rhinitis, asthma and so on (Lu et al., 2012; Li et al., 2020; Weidner et al., 2021). miRNAs play important roles in different physiological and pathological processes of nervous system diseases, neurovascular diseases, and diabetes (Vienberg et al., 2017; Sun et al., 2018; Gasecka et al., 2020; van den Berg et al., 2020; He et al., 2021; Wang, 2021; Wang L. et al., 2021; Su et al., 2022). They have become a biomarker of cardiovascular diseases, ischemic stroke, and neurodegenerative diseases because of their regulation of gene expression (Eyileten et al., 2021). miRNAs have been shown to be consistently maintain in the blood and cerebrospinal fluid of patients with Alzheimer’s disease (AD), are related to AD-related proteins in the brain, and play a role in the pathogenesis of AD (Qiu et al., 2015; Takousis et al., 2019). miRNAs are related to multiple pathophysiological pathways in Parkinson’s disease and target the genes BCL2, BDNF, and SIRT1 by upregulating miR-9, miR-34a, and miR-141 (Rostamian Delavar et al., 2018). miR-939 and miR-26a are related to the neuroinflammatory response and oxidative stress in the pathogenesis of Parkinson’s disease (Martinez and Peplow, 2017). In ischemic stroke, reduced ischemia is associated with the downregulation of miR-30a, which is achieved by enhancing Beclin-1-mediated autophagy (Wang et al., 2014). Upregulation of miR-146a is associated with neuroprotection in cerebral ischemia (Zhou X. et al., 2016; Figure 2).

## 3. The role of miRNAs in the pathogenesis of SCI

Numerous studies have confirmed the role of miRNAs in SCI secondary injury (inflammatory response, angiogenesis, axon regeneration, glial cell development, etc.; Strickland et al., 2011; Yunta et al., 2012; Bhalala et al., 2013; Hu et al., 2013; Shi et al., 2017). miRNAs regulate the expression of related proteins by up-regulating or down-regulating target genes that are altered after SCI. For example, the changes of miR-10a, miR-10b, miR-142-3p, miR-338 and miR-133 contents after SCI, which are closely related to the pathogenesis of the disease (Zhou S. et al., 2016). Exosomes derived from bone marrow mesenchymal stem cells have been shown to inhibit the NF-κB pathway by upregulating miR-23b targeting TLR4, participate in the process of oxidative stress, alleviate the inflammatory response after SCI, and improve motor function of rats after SCI (Nie and Jiang, 2021). By pretreating neuron-derived exosomes rich in miR-126-3p under hypoxic conditions, PIK3R2 can be regulated to reduce pain hypersensitivity induced by ischemia–reperfusion injury (Wang H. et al., 2021). miR-29a/199B inhibits the RGMA/STAT3 axis and promotes neural function repair in rats after SCI (Yang and Sun, 2020). Neuron-derived exosomes regulate astrocyte and microglia activation through miR-124-3p to protect against traumatic SCI (Jiang et al., 2020). Intravenous administration of miR-133b decreased macrophage aggregation and extracellular matrix protein expression at the site of SCI and reduced harmful fibrous scar formation after SCI (Theis et al., 2017). Overexpression of miR-223 decreased the protein expression levels of interleukin (IL)-1β, IL-18, NLRP3, ASC, and caspase-1, and regulated the transformation of macrophages between types in injured spinal cords of mice with chronic sciatic nerve injury (Zhu et al., 2021). In addition, miR-20a, miR-21, miR-497, miR-494, miR-223, miR-29b, miR-320, and miR-124 were involved in the apoptosis of cells after SCI (Jee et al., 2012b; He et al., 2015; Zhao et al., 2015; Xu et al., 2016; Bai et al., 2021; Huang et al., 2021; Malvandi et al., 2022; Ma et al., 2022a). miR-133b, miR-20a, and miR-124 are involved in promoting angiogenesis and regulating nerve repair after SCI (Yu et al., 2015; Theis et al., 2017; Cui et al., 2019; Wang T. et al., 2019; Danilov et al., 2020).

### 3.1. Mechanisms of miRNA in neuropathic pain after SCI

**Figure 3:**
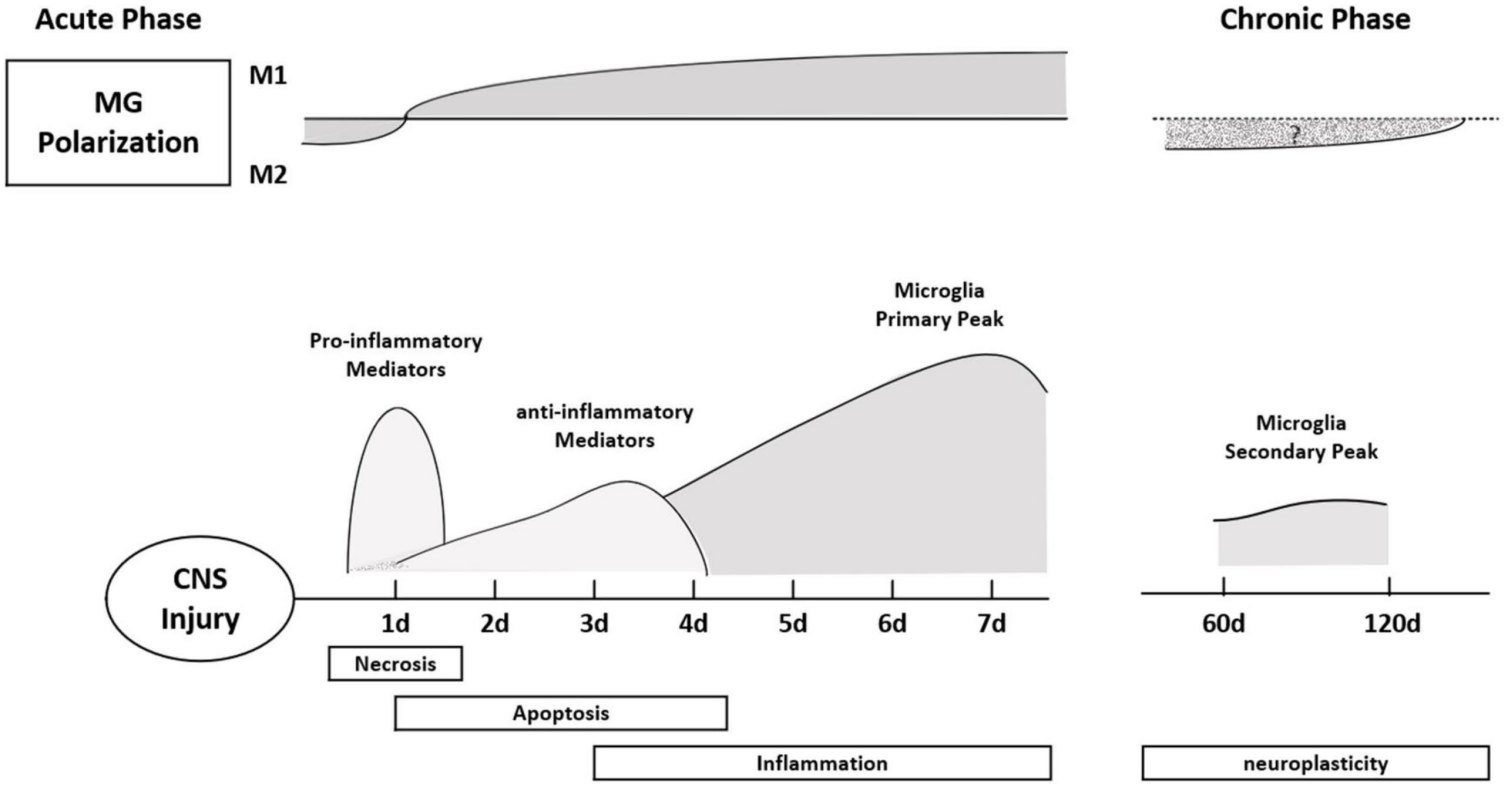
Polarization typing of microglia at different times after SCI. In the acute phase, microglia mainly express M1, showing a pro-inflammatory response. The pro-inflammatory effect of microglia reached a peak on the seventh day after the injury, and the inflammatory response became more obvious; in the chronic phase, the M1 type transformed into the M2 type, showing an anti-inflammatory effect. Finally, the second peak of microglia appeared during the neural remodeling phase (6–8 weeks after SCI).

As a protective response mechanism of the body, pain often indicates that some tissues are in or are about to be in a state of injury. Increasing evidence suggests that miRNA expression, DNA methylation, and histone modifications are associated with chronic pain (Descalzi et al., 2015). Chronic pain is accompanied by cognitive, emotional, and anxiety disorders. Microglia and astrocytes, as glial cells in the spinal cord, play an important role in neuroinflammation and nerve conduction to regulate pain (McWilliams et al., 2003). Microglia are the resident immune cells in the central nervous system. In the acute inflammatory phase of SCI, M1 microglia are mainly seen, causing a pro-inflammatory response. In the chronic phase of SCI, M1 microglia transform to M2 microglia in the secondary injury phase, showing anti-inflammatory effects (Figure 3). miRNAs are considered to be “genetic switches” for microglial transformation between phenotypes. With the progress of sequencing technology, the expression profiles of miRNAs in microglia have been gradually identified. Most miRNAs can target mRNAs to downregulate protein expression in microglia and prevent the progression of neuropathic pain (Jeong et al., 2016; Inoue and Tsuda, 2018). miRNAs and other non-coding RNAs are considered key in the pathogenesis of the inflammatory response, nerve injury, and pain, and are seen as potential therapeutic targets (Lu and Rothenberg, 2018; Ling et al., 2021) and play an important role in the occurrence and development of inflammation (Bai et al., 2007). Non-coding RNAs have been widely studied and are regarded as key factors involved in the pathophysiology of chronic pain, and their main mode of action is the deletion of Dicer (an enzyme involved in miRNA production). In rats with Dicer1 gene knockdown, the expression of the glial fibrillary acidic protein was inhibited by miR-17-5p, and the proliferation of astrocytes was inhibited (Hong et al., 2014). miRNAs play a regulatory role in nociceptive hypersensitivity (Schinkel et al., 2006; Sakai and Suzuki, 2015) and affect neuronal excitability by changing the expression of ion channels (Wang, 2013). Cav1CaV1.2l-type calcium channel underlies plasticity in chronic neuralgia. As a direct target of caav1.2l-type calcium channel, miR-103 can induce neuropathic pain by intrathecal injection of miR-103 (Fossat et al., 2010; Favereaux et al., 2011). Spinal cord injury not only affects the expression of miRNA in dorsal root ganglion (DRG) neurons but also affects the expression of miRNA in other neurons and glial cells in the spinal cord and brain. Cells at the site of injury are not directly affected but can function through glial activation and remodeling of synaptic plasticity. Neurons of the DRG and trigeminal ganglion, as first-line nociceptive signalers, are the primary sensory neurons in SCI. Extracellular miRNAs can activate DRG neurons, generate rapid inward currents, and mediate nociceptive sensation with the help of Toll-like receptors (Park et al., 2014). The expression of miR-155 changes in inflammation-related diseases, and it is upregulated after osteoarthritis. Intrathecal injection of miR-155 inhibitors attenuates neuropathic pain, inhibiting the corresponding proinflammatory cytokines. miR-195 has been reported to mediate the neuroinflammatory response and neuropathic pain by regulating autophagy, a key component of neuroinflammation after SCI (Li X. et al., 2013; Vigorito et al., 2013; Tan et al., 2015). ATG14 is a key regulator in the process of autophagy and a direct target of miR-195; by upregulating miR-195, the autophagy response is inhibited, and the neuroinflammatory response is enhanced, intensifying neuropathic pain (Obara and Ohsumi, 2011; Shi et al., 2013). miR-203 and miR-124 are also involved in the neuroinflammatory response after SCI. The target of the former is the Ras-related protein Rap-1A(RAP1A)3′-UTR. The content of RAP1A in the dorsal spinal cord increases after spinal ganglion shock. In previous studies, it has been shown that the expression of miR-203 was increased in the spinal cord after formalin stimulation, which also indicated that mir-203 may be involved in neuropathic pain (Urayama et al., 1997; Li H. et al., 2015). Intermittent electrical stimulation could reduce the expression of miR-124 in rats after SCI, and intrathecal injection of miR-124 could reduce neuropathic pain caused by peripheral nerve injury. Therefore, miR-124 may also be involved in the occurrence and development of neuropathic pain after SCI (Strickland et al., 2014). Different types of disease have quantified abnormal miRNA expression in body fluids and biopsied tissues, linking this abnormal expression to inflammation and pain (Andersen et al., 2014, 2016; Lin et al., 2017). Studies have mainly focused on miR-21, miR-146a, and miR-155 (Tafuri et al., 2015). It is necessary and meaningful to study the mechanisms the involvement of miRNA in neuropathic pain after SCI, which can not only provide a diagnostic basis for treatment of patients with pain but can also provide evidence for disease prognosis.

### 3.2. miRNAs in neuronal repair and axon regeneration after SCI

miRNAs are tightly controlled during neural development and can regulate the expression of multiple genes. According to a study from Li et al., miRNAs and related signaling pathways play important roles in the formation of glial scars and axonal regeneration after SCI (Li et al., 2016), including neuronal degeneration and remodeling, axonal regeneration, mRNA degradation, and myelin reformation caused by translation inhibition (Fiorenza and Barco, 2016). The proliferation of astrocytes, the formation of myelin debris, and the appearance of scar tissue are the causes of axonal regeneration and nerve repair disorders after SCI, which play a decisive role in recovery (Zheng et al., 2012). Regulatory factors such as mTOR, STAT3, NF-kB, cAMP, and JNK are related to the regulation of astrocytes in disease pathogenesis (Hung et al., 2016). The effects of miRNAs on astrogliosis through the regulation of signaling pathways has been gradually confirmed. For example, miR-590 and miR-582 can target NF-kB signaling; miR-146a, miR-133b, and miR-124 can target the regulation of the RhoA signaling pathway (Li P. et al., 2015; Xu et al., 2015). Targeting miR-21 may serve as a therapeutic approach to control gliosis after SCI and improve the prognosis of the disease (Sahni et al., 2010). Overexpression of miR-145 can reduce the number of astrocyte-related cell processes and affect cell proliferation and migration (Wang et al., 2015). miRNAs may be directly or indirectly involved in the repair of nerve injury caused by SCI. The dramatic changes of miRNAs in axonal regeneration after SCI also suggest that miRNAs play a key role in the pathogenesis of SCI (Bareyre and Schwab, 2003; Nakanishi et al., 2010). miRNAs increase in abundance after SCI and are regulated by SCI; abnormal miRNA expression may aggravate SCI. A bioinformatics analysis showed that abnormally expressed miRNAs play regulatory roles by acting on potential targets, and several miRNAs have been identified to be involved in axonal regeneration. RhoA is an inhibitor of axon growth. In non-mammalian zebrafish, miRNA-133b is upregulated by the small molecule GTPase RhoA to regulate protein levels to restore motor function and promote axon regeneration (Yu et al., 2012). In recent years, more and more studies have mainly focused on the role of miRNAs and the Rho/ROCK pathway in axonal growth promotion and neuronal apoptosis inhibition (Yan et al., 2012; Li et al., 2016; Zhou S. et al., 2016). In mouse P19 cells, overexpression of miR-124 can promote axon growth, and research shows that blocking miR-124 inhibits axon growth (Yu et al., 2008). miR-124 can also reduce the SCI area through the reduction of astrocytes and increasing neurons (Xu et al., 2012). In addition, miR-138 regulates axonal regeneration through negative feedback by interacting with SIRT1, a histone deacetylase dependent on its target, NAD (Liu et al., 2013; Li X. H. et al., 2013). Overexpression of miR-20a in damaged spinal cord tissues led to sustained degeneration of motor neurons when knocked out in a study by Jee et al. (2012a,b). The corresponding target gene protein levels increased after miR-20a overexpression, reducing apoptosis and improving motor function (Jee et al., 2012b) Increased expression of miR-486 also increased oxidative stress-mediated neurodegenerative responses (Jee et al., 2012a).

### 3.3. Role of miRNA in vascular regeneration after SCI

Neurogenesis and angiogenesis are closely related and often occur synchronously after SCI. Endothelial cells regulate neuronal differentiation through the secretion of soluble factors *in vitro* (Fuchs et al., 2004; Ohab et al., 2006). Promoting angiogenesis and neurogenesis is beneficial to rehabilitation after SCI and stroke, and they are regulated by multiple miRNAs. miR-27a regulates the TLR4 signaling pathway by downregulating TICAM-2, reduces reperfusion injury in rats after SCI, reduces the inflammatory destruction of the blood–spinal cord barrier caused by spinal cord ischemia, and reduces edema (Li X. Q. et al., 2015). Mesenchymal stem cell-derived exosomes transfected with miR-126 promoted neurogenesis and angiogenesis by inhibiting cell apoptosis and restoring neural function after SCI (Huang et al., 2020). miR-21 is related to vascular endothelial growth factor, the Ang-1 receptor, and Ang-1 after SCI (Ge et al., 2014). Locally permeable macrophages after SCI accelerate mitochondrial damage by delivering miR-155, which activates the NF-κB signaling pathway by targeting the suppressor of cytokine signaling 6 (SOCS6) and inhibits p65 ubiquitination and degradation. This study demonstrated the role of the miR155/SOCS6/p65 axis in regulating the mitochondrial function of vascular endothelial cells and attempted to describe the causes of crosstalk and the mechanisms of interaction between vascular endothelial cells and macrophages after SCI (Ge et al., 2021). miR-210 inhibits ePhrin-A3 and protein tyrosine phosphatase to promote angiogenesis after SCI (Ujigo et al., 2014). miR-223 is also involved in post-SCI angiogenesis (Liu et al., 2015). miR-107 increases endothelial VEGF165/164 levels and the number of capillaries in ischemic stroke (Li Y. et al., 2015).

### 3.4. miRNAs may be biomarkers for SCI

**Figure 4:**
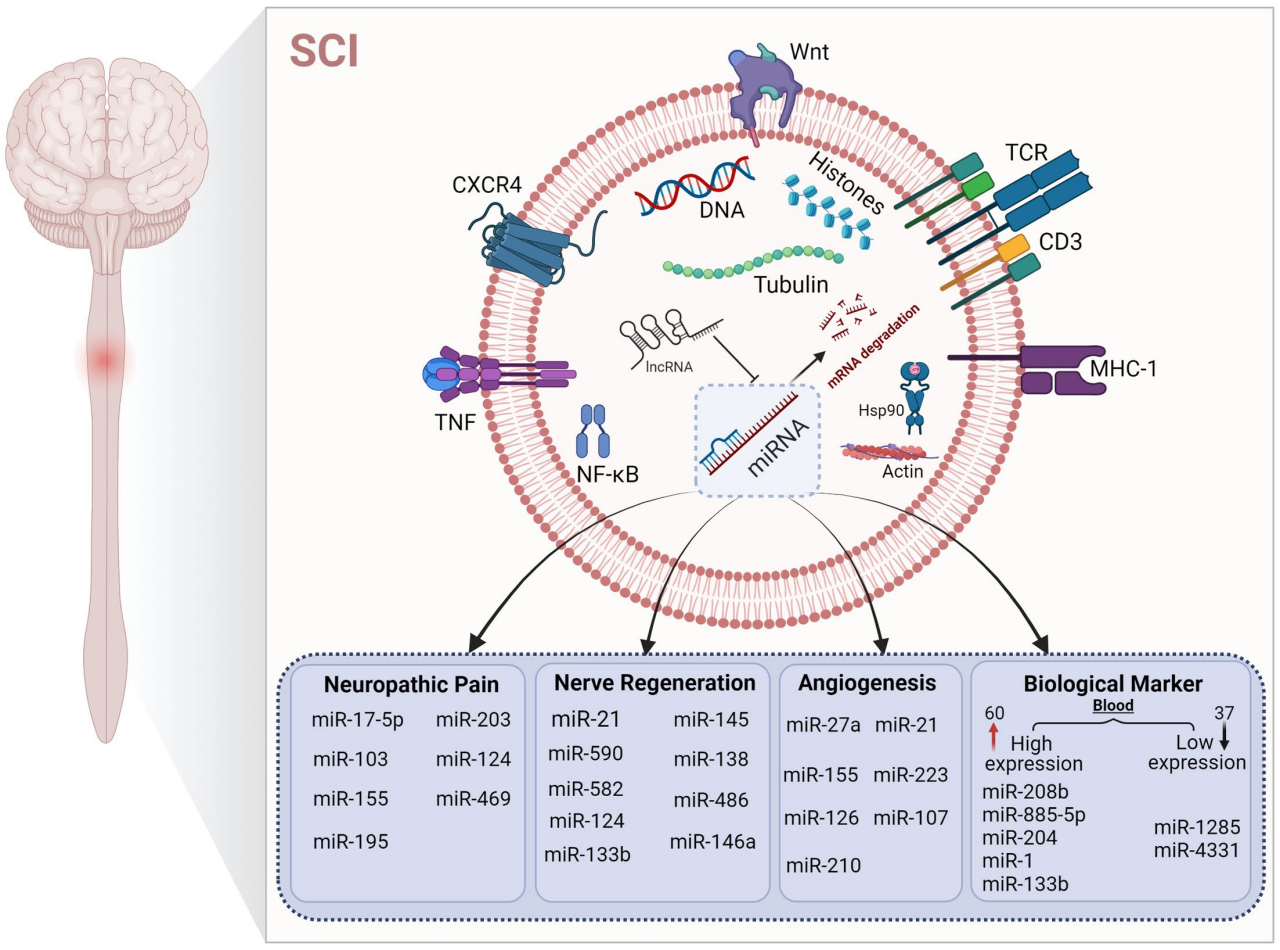
Regulation of microRNAs in different mechanisms after SCI. The figure shows that microRNAs play a key role in different pathological states after SCI. The up-regulation and down-regulation of microRNA content affect various pathophysiological processes to varying degrees. Under the regulation of microRNA, mRNA expression is affected, which further affects the expression of corresponding target proteins.

miRNAs have two major characteristics of tissue specificity and stability in body fluids, and changes in miRNA content after SCI is a prerequisite for their possible role as a post-SCI biomarker. At present, the gold standard for clinical diagnosis of SCI is MRI and the American Spinal Injury Association Impairment Scale, but due to the poor basic condition of patients, comorbidity with other injuries, and the limited level of drug and surgical treatment availability, the reliability of these two assessments decreases (Lubieniecka et al., 2011). Proteins can be used as biomarkers; however, despite extensive studies in animal models, no standard treatments have been established based on protein biomarkers. It is difficult to determine the severity of related symptoms and variability of injury recovery caused by secondary injury after SCI solely by neurological examination (Yang et al., 2018). Glial fibrillary acidic protein and calcium-binding protein S100-β are potential protein biomarkers of astrocytes in injured tissues, and the levels of these two proteins in cerebrospinal fluid and serum of patients with SCI are higher than in healthy controls (Kwon et al., 2010). Proteins are sometimes collected from cerebrospinal fluid to determine the location and extent of the damage, and cerebrospinal fluid is harvested mainly by invasive lumbar puncture. The operation is difficult, and patient acceptance is low. Therefore, to quickly determine the scope of SCI, finding specific biomarkers that can be directly derived from blood or body fluids is more conducive to clinical practice and precisely targeted interventions. Recent studies have shown that miRNA is tissue-specific and stable in body fluids, making it a good candidate as a blood biomarker (Laterza et al., 2009). About 400 miRNAs have been identified using microarrays, and nearly 300 of them showed altered expression in rat models of SCI, with mutations in 97 miRNAs, including 60 with increased expression and 37 with decreased expression (Jin et al., 2014; Ning et al., 2014). Jee et al. upregulated the expression of NeuroD6 and Ngn1 and reduced apoptosis by inhibiting miR486 and miR20a and were the first to use miRNAs as novel drug targets for the treatment of human SCI (Jee et al., 2012a,b). Expression of miR-181a, miR-127, miR-1, miR-206, miR-152, miR-221, and miR-214 was changed after SCI, among which miR-181a and miR-127 affected the expression levels of CplA2 and SPLA2 in the cytosol. miR-1, miR-206, miR-152, miR-221, and miR-214 are involved in the pathogenesis of SCI *via* gene regulation on corresponding targets such as intercellular adhesion molecule 1, tumor necrosis factor-α and IL-1β (Baichurina et al., 2021). Tigchelaar et al. studied the expression of miRNAs in pig serum and cerebrospinal fluid in animals with different degrees of SCI. The results showed that two miRNAs (miR-1,285, miR-4,331) were decreased in pig serum, while the other five miRNAs (miR-208b, miR-885-5p, miR-133b, miR-204, and miR-1) were increased. This indicates that the total miRNAs in serum are correlated with the degree of SCI, which further indicates that miRNAs may serve as biological markers and have a certain guiding value in SCI (Tigchelaar et al., 2017). Based on this study, Tigchelaar et al. further observed the distribution of miRNAs by collecting cerebrospinal fluid and blood from patients with clinical acute SCI. The results showed significant changes in 50% of miRNAs in patients with SCI, with the greatest changes (at least 190) in the levels of miRNAs detected in cerebrospinal fluid (CSF). Different from the above results, experiments in non-human animals showed that serum miRNAs were more strongly correlated with the severity of SCI and the prognosis of the disease (Tigchelaar et al., 2019). miRNAs, as epigenetic participants, are common markers of disease, which also makes them key regulators in various pathophysiological processes. In addition to miRNA, non-coding RNAs, such as long-and short-ncRNAs and piRNA, may also affect the occurrence and development of human diseases. Abnormal regulation of miRNAs occurs in cellular processes and disease, and changes in miRNA levels can be detected in body fluids, making them potential targets for clinical treatment and diagnostic markers, which are helpful for disease classification and diagnosis (Nieto-Diaz et al., 2014; Yu et al., 2015; Shi et al., 2017; Figure 4).

### 3.5. SCI therapy based on miRNAs

The neuroprotective effects based on microRNAs in SCI experiments are mainly reflected in the following aspects. (1) Using lentiviral transfection technology to reduce lesion volume and improve motor function after SCI by targeted upregulation of a specific microRNA (miR-124; Gu et al., 2017), adeno-associated virus-miR-383-infected bone marrow infused locally with bone marrow interstitial stem cells was able to help restore motor function in rats while protecting tissue integrity (Wei et al., 2017). (2) miR-210 improved motor function after SCI by acting on astrocytes and vascular microcirculation (Zhu et al., 2017). (3) Given the role of microRNAs in neuroprotection and inflammatory responses after SCI, miR-27a has also been used to improve treatment and research protocols for blood–brain barrier protection after spinal cord ischemia–reperfusion injury (Li X. Q. et al., 2015). (4) The use of miRNA inhibitors plays a significant role in reducing secondary injuries after SCI. The selection of treatment methods often starts with miRNA inhibitors to observe their effects on neurogenesis and neuronal survival. Animal studies have confirmed that miR-20a inhibitors, miR-486 inhibitors, and miR-223 inhibitors could improve hindlimb motor function in SCI (Guan et al., 2019; Wang T. et al., 2019; Wang Y. et al., 2019). In a rat model of transient spinal cord ischemia, inhibition of miR-320 significantly improved the motor function of the hind limbs. This process may be caused by an increase in phosphorylated Hsp20 content. Inhibiting the expression of miR-320 can not only play a neuroprotective role but also prevent the exacerbation of ischemia reperfusion injury (He et al., 2015).

## 4. Circrnas and microRNAs in SCI

CircRNAs are important immunoreactive elements in regulating disease-related pathophysiological environment and gene expression. It has a stable loop structure in the mammalian cytoplasm and has a very rich binding site for microRNAs. CircRNAs have sequence specificity and structure specificity. More and more evidence shows that circRNAs have 5′ cap and 3′ tail structures, and generate covalent closed-loop structure through reverse splicing (Bagchi, 2018; Wu et al., 2019), that is, phagocytic microRNAs by acting as a microRNAs sponge. Competing endogenous RNA (ceRNA) is involved in gene expression and regulating transcription, and interacts with microRNA to play an important biological and regulatory role in the progression of disease (Ma et al., 2022b). Especially in SCI it can activate astrocytes, improve neuroinflammatory response and regulate neuronal apoptosis, and participate in the regulation of nerve repair and regeneration. The increasing expression of circRNA during neuronal differentiation suggests that circRNA plays an important role in neurological diseases. It has been shown that the circRNA-01477/miR-423-5p axis plays an important role in the regeneration microenvironment after SCI. In addition, the expression of circRNAs can be displayed more directly through high resolution *in situ* hybridization, and the dynamic influence of circRNAs on synaptic function can be reflected (You et al., 2015; Peng et al., 2021). Using whole transcriptome sequencing, abnormal circRNAs and microRNAs were identified 3 days after SCI. It was also determined that miR-223-3p, miR-182, circRNA-003801, circRNA-014620 and circRNA-013613 may be related to the inflammatory response after SCI (Peng et al., 2020). In order to elaborate the relationship between circRNAs and microRNAs, relevant studies have also formed circRNA-microRNA-mRNA networks through gene expression profile construction and gene chip technology, further clarifying the interaction between some specific circRNAs and microRNAs (Sámano et al., 2021). In conclusion, the role of circRNAs in SCI cannot be ignored. In SCI, circRNAs are closely related to microRNAs, and both participate in gene expression regulation and protein expression translation.

## 5. Challenges and perspectives of miRNAs

Secondary injury after SCI often leads to changes in the immune microenvironment of the spinal cord and then changes the expression content of miRNAs *in vivo*. Previous studies on miRNAs have shown that they play an important role in supporting neuronal survival and providing plastic conditions for neural regeneration. However, due to “off-target effects” caused by the rapid degradation of abundant RNases in circulation or during cell phagocytosis and the interactions between miRNAs and target genes, the accurate delivery of miRNAs has become an urgent problem to be solved. Multiple and repeated injections may improve this accordingly. This requires us to further develop the study of miRNAs to identify specific target genes and block off-target effects. In addition, different responses of miRNAs in the brains of male and female mice after focal cerebral ischemia were found to affect experimental results, and female mice have been suggested to be included in experiments to overcome sex bias in research. Research has suggested that miRNAs are greatly influenced by sex, contributing to the risk and limitations of clinical translation of miRNAs in SCI treatment. Most studies have focused on the regulation of a single miRNA as a therapeutic target, and current studies focus on broad-spectrum analysis of all miRNAs using bioinformatics methods. Although analysis software has predicted miRNA–target gene interactions in broad spectrum studies, the specific interactions of many miRNAs have not been further verified using experimental inhibition or knockdown.

Despite many current challenges and limitations, miRNA-based therapy at the molecular level has emerged as an effective strategy for the treatment of central nervous system injury. To try an explore how miRNAs affect different target genes and screen relevant candidate miRNAs for disease treatment, studies should focus on developing reasonable and effective miRNA dosages and forms and find accurate drug delivery methods to reduce and block off-target effects. The discovery of such mechanisms is necessary for the development of clinically effective miRNA-based drugs, and a large number of clinical trials are needed to confirm the clinical significance and application value of miRNA therapy.

## Author contributions

CZ, JL, and YY: design and concepts. YY, JL, and CZ: definition of intellectual content. CZ, ZT, XX, WL, HK, YP, and ZeL: literature search. CZ, ZT, XX, YL, FB, and YJ: data acquisition. CZ: manuscript preparation. CZ, YL, and ZiL: manuscript editing. JL, YY, DY, LD, and FG: manuscript review. All authors contributed to the article and approved the submitted version.

## Funding

This work was funded by the Fundamental Research Funds for Central Public Welfare Research Institutes (2022CZ-3) and the National Natural Science Foundation of China (general program 81870979 and 82071400).

## Conflict of interest

The authors declare that the research was conducted in the absence of any commercial or financial relationships that could be construed as a potential conflict of interest.

## Publisher’s note

All claims expressed in this article are solely those of the authors and do not necessarily represent those of their affiliated organizations, or those of the publisher, the editors and the reviewers. Any product that may be evaluated in this article, or claim that may be made by its manufacturer, is not guaranteed or endorsed by the publisher.
